# Latent profiles and influencing factors of sleep quality in patients with chronic obstructive pulmonary disease

**DOI:** 10.3389/fmed.2025.1692930

**Published:** 2025-10-27

**Authors:** Jing Li, Na Zhou, Xinxia Li

**Affiliations:** ^1^School of Nursing, Inner Mongolia Medical University, Hohhot, China; ^2^Inpatient Department, Affiliated Hospital of Inner Mongolia Medical University, Hohhot, China

**Keywords:** COPD, sleep quality, latent profiles, influencing factors, sleep

## Abstract

**Objective:**

This study aims to elucidate the classification characteristics and determinants of sleep quality in individuals with chronic obstructive pulmonary disease (COPD) to inform tailored interventions.

**Methods:**

A total of 231 COPD patients were selected using convenience sampling from January to June 2025 at the respiratory and critical care department of a tertiary hospital in Inner Mongolia. Data were collected through a general questionnaire, Pittsburgh Sleep Quality Index, COPD Assessment Test, and Self-Efficacy for Managing Chronic Disease 6-Item Scale. Latent profile analysis was employed to categorize sleep quality, and logistic regression was conducted to identify factors influencing each profile.

**Results:**

Sleep quality among COPD patients exhibited three distinct categories: better sleep group (40.6%), low efficiency-maintenance group (46%), and high hypnotic-use with daytime dysfunction group (13.4%). Sex, COPD Assessment Test score, and self-efficacy score were found to influence sleep quality in COPD patients.

**Conclusion:**

There is population heterogeneity in sleep quality among patients with COPD. Special attention should be given to individuals with low efficiency-maintenance and high hypnotic-use with daytime dysfunction groups to implement targeted interventions aimed at enhancing sleep satisfaction and overall quality of life.

## 1 Introduction

Chronic obstructive pulmonary disease (COPD) is a diverse pulmonary condition characterized by persistent respiratory symptoms like dyspnea, cough, and sputum production resulting from airway and/or alveolar abnormalities, leading to airflow limitation. Epidemiological data indicates a global COPD prevalence of 10.3%, with an estimated 600 million affected individuals projected by 2050 (1). In China, nearly 100 million people are afflicted by COPD, with 21.2% of them aged over 60 (2), thus confronting a substantial disease burden. Concurrently, sleep disturbances are highly prevalent among COPD patients, with reported rates ranging from 40 to 75% (3). These disturbances manifest as difficulties initiating and maintaining sleep, along with early morning awakenings, significantly impacting patients’ daytime functioning. Given that COPD patients dedicate a considerable portion of their lives to sleep, disruptions in normal sleep patterns may contribute to adverse long-term outcomes. Furthermore, poor sleep quality can influence the progression and prognosis of COPD. Omachi et al. (4) demonstrated that insomnia is associated with a 4.7-fold increase in COPD exacerbation risk and heightened susceptibility to symptoms like fatigue, depression, and diminished quality of life (5). Sleep disorders in COPD patients result from various factors, with a key factor being reduced auxiliary respiratory muscle tone during rapid eye movement sleep, leading to increased reliance on diaphragmatic breathing. Additionally, airway obstruction, hypoxia, and hypercapnia resulting from the disease itself contribute to sleep disturbances by disrupting respiratory function. Furthermore, medications such as β2 receptor agonists and glucocorticoids can induce side effects like palpitations and insomnia (6). Psycho-social factors also play a role in influencing sleep patterns, making sleep issues unique to each COPD patient.

Current research on sleep disorders in COPD patients often overlooks potential subgroup differences, hindering the identification of distinct patient characteristics related to sleep problems. This limitation impacts the specificity and efficacy of clinical interventions. Latent profiling, a human-centered statistical method, offers a solution by revealing hidden subgroups within the COPD patient population based on various observational metrics. This approach provides a more comprehensive understanding of the sleep challenges encountered by COPD patients, offering insights that can improve the tailoring of interventions for better outcomes.

## 2 Object and method

### 2.1 Object of study

COPD patients were selected using a convenience sampling method between January and June 2025 from the respiratory and critical care department of a hospital in Inner Mongolia Autonomous Region. The inclusion criteria were as follows: (1) meeting the diagnostic criteria outlined by the Global Initiative for Chronic Obstructive Pulmonary Disease 2025 (1); (2) being 18 years of age or older; (3) demonstrating willingness to participate in the study by signing an informed consent form. Exclusion criteria included: (1) unconsciousness or inability to communicate effectively; (2) presence of mental illness, hearing impairment, or cognitive impairment that would hinder questionnaire completion. The sample size was determined using Kendall’s method, which suggests that the sample size should be 5–10 times the number of predictor variables. With 22 independent variables in this study, accounting for a 20% potential invalid response rate, the required sample size ranged from 132 to 264 cases. Ultimately, 231 cases were included in the study. This study has been approved by the Ethics Committee of Inner Mongolia Medical University Affiliated Hospital (No. KY2025086) and strictly conducted in accordance with the Declaration of Helsinki.

### 2.2 Survey instruments

#### 2.2.1 Self-made questionnaire

Self-made questionnaire collected patients’ gender, age, residence, occupation, education level, marital status, medical insurance type, family income per capita, smoking history, drinking history, diagnosed COPD years.

#### 2.2.2 Pittsburgh Sleep Quality Index (PSQI)

Pittsburgh Sleep Quality Index (PSQI) compiled by Dr. Buysse (7), a psychiatrist at the University of Pittsburgh, there are seven dimensions: subjective sleep quality, sleep latency, sleep duration, sleep efficiency, sleep disturbances, use of sleeping medications and daytime dysfunction. The total score is 0 ~ 21 points, and the higher the score, the worse the sleep quality. The Chinese version of PSQI Cronbach’s *α* coefficient is 0.82–0.83 (8).

#### 2.2.3 COPD assessment test (CAT)

The scale assesses the influence of COPD symptoms on health and daily activities through eight items: cough, expectoration, chest discomfort, sleep, energy, mood, exercise capacity, and daily physical activity. Each rated on a scale of 0 to 5 points, resulting in a total score ranging from 0 to 40 points. A higher score indicates a more significant impact of COPD on patients. The scale demonstrates a high internal consistency with a Cronbach’s alpha coefficient of 0.88 (9). In this study, the Cronbach’s alpha coefficient of the scale was 0.748, and our data confirms the robustness of the scale.

#### 2.2.4 Self-Efficacy for Managing Chronic Disease 6-Item Scale (SEMCD6)

Self-Efficacy for Managing Chronic Disease 6-Item Scale used to evaluate the self-management behavior and ability of chronic disease patients (10), consisting of two dimensions. Symptom management self-efficacy (items 1–4) and disease common management self-efficacy (items 5–6) are assessed using a visual simulation method, with scores ranging from 0 to 10. A score of 1 indicates “no confidence,” while 10 indicates “full confidence.” The average score is computed, with a cutoff value of 5 points. Scores ≤5 indicate low self-efficacy, 5–7 points indicate medium self-efficacy, and scores ≥7 indicate high self-efficacy, demonstrating a Cronbach’s *α* coefficient of 0.87 (11). In our sample, the Cronbach’s alpha coefficient of the scale is 0.92.

### 2.3 Data collection methods

A paper questionnaire was administered to patients on their first day of admission after providing an initial explanation of the study’s objectives. Following this, participants provided their informed consent by signing a consent form. Any queries raised by patients during the questionnaire completion process were addressed by the investigator. Subsequently, the investigator conducted an immediate review to identify and rectify any missing information in the completed questionnaires.

### 2.4 Statistical method

Statistical analysis was conducted using SPSS 27 software. The data were assessed for normal distribution, and categorical data were summarized using frequency and percentage. Statistical inference was performed utilizing the chi-square test, Fisher’s exact test, one-way ANOVA, Kruskal-Wallis H test, and unordered multiclass logistic regression. A significance level of *p* < 0.05 was applied to determine statistical significance. The selection of unordered multiclass logistic regression was based on specific considerations. Firstly, the primary aim of this study was to investigate the impact of various variables on the likelihood of individuals belonging to different categories. As the dependent variable is an unordered multiclass nominal variable, unordered multiclass logistic regression is the most appropriate method, providing easily interpretable odds ratios. Alternative methods were considered and ruled out for the following reasons. Linear regression necessitates continuous and evenly spaced dependent variables, which do not align with our discrete and non-sequential dependent variables. Utilizing linear regression in this context would violate its underlying assumptions and yield inaccurate results. Ordered logistic regression requires a hierarchical order among dependent variable categories, which is not theoretically or statistically supported in our case. Imposing ordered models in the absence of such hierarchy could distort the relationship between variables and outcomes. Mplus8 was utilized to delineate distinct profiles of seven dimensions of sleep quality among COPD patients. The number of categories was systematically incremented from one until achieving the optimal model fit. Model fit was assessed using various indicators including Akaike information criterion (AIC), Bayesian information criterion (BIC), adjusted BIC (aBIC), Entropy, Bootstrapped Likelihood Ratio Test (BLRT), and Lo–Mendell–Rubin Test (LMRT). Smaller values of AIC, BIC, and adjusted BIC indicate superior model fit, while an Entropy value closer to 1 signifies higher classification accuracy. A significance level of *p* < 0.05 for BLRT and LMRT indicates that the model with K classes is a better fit than the model with K-1 classes (12).

## 3 Result

### 3.1 Latent profile analysis of sleep quality in COPD patients

The seven dimensions of the Pittsburgh Sleep Quality Index (PSQI) were utilized as explicit variables for potential profile analysis, as shown in Table 1 for analysis indicators. Scores for these dimensions are treated as continuous variables and follow a normal distribution, estimated using maximum likelihood estimation. To ensure global optimality of the model solution rather than local optimality, random initial values were assigned to each model multiple times using the instruction STARTS = 100,250. Furthermore, upon questionnaire completion, the investigator promptly checked for any missing items on-site and reminded participants to provide any omitted information, resulting in a dataset without missing values. The LMRT and BLRT for Models 2 and 3 yielded statistically significant results, as presented in Table 1 (*p* < 0.05). Model 3 exhibited a significant decrease in AIC, BIC, and aBIC values compared to Model 2, indicating its superior fit to the data. The entropy value of Model 3, which was 0.951, exceeded the threshold of 0.8, suggesting improved classification accuracy, with each category representing more than 5% of the total sample. Consequently, a three-category solution was identified as the most appropriate model.

**Table 1:** Potential profile indicators of sleep quality in COPD patients.

Model	AIC	BIC	aBIC	LMRT	BLRT	Entropy	Class probability (%)
1	4243.596	4291.79	4247.418	–	–	–	–
2	3816.483	3892.216	3822.489	0	0	0.984	47.2/52.8
3	3516.027	3619.3	3524.217	0.0079	0	0.951	40.6/46/13.4
4	3373.555	3504.367	3383.929	0.2809	0	0.962	8.2/37.2/44.6/9.9

**Figure 1:**
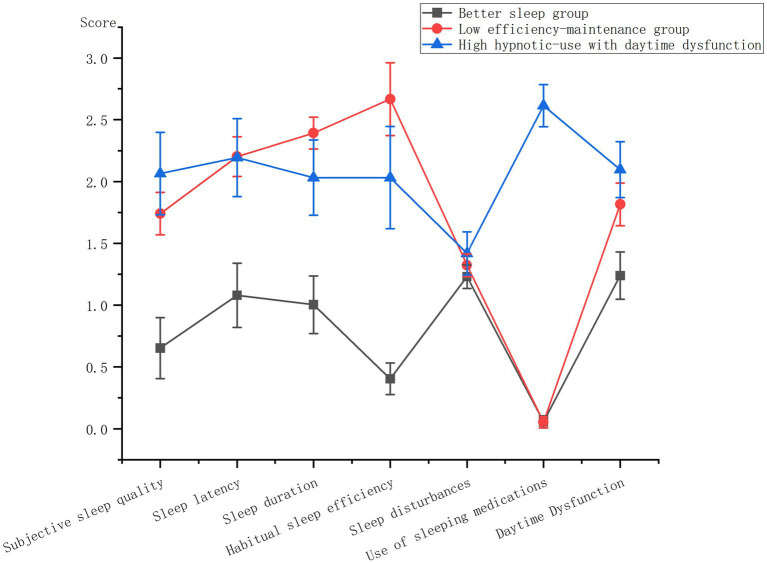
Differences in scores of sleep quality dimensions in COPD patients with different potential profiles.

Subsequently, the mean scores of different potential profiles on each sleep quality dimension among COPD patients were graphically represented in Figure 1. Class 1 demonstrated significantly lower scores across all dimensions except for the use of hypnotic drugs, leading to its designation as the “better sleep group.” Class 2 exhibited moderate sleep quality, with the poorest sleep efficiency and shortest sleep duration, earning it the label of “low efficiency-maintenance group.” Lastly, Class 3 displayed the highest frequency of hypnotic drug usage and the most pronounced daytime dysfunction, hence being denoted as the “high hypnotic-use with daytime dysfunction group.”

### 3.2 Single factor analysis of sleep quality categories in COPD patients

Table 2 displays statistically significant variations in gender, age, physical activity, CAT score, and self-efficacy score across distinct sleep quality profiles in COPD patients (*p* < 0.05).

**Table 2:** Comparison of distribution of different variables in sleep quality categories of COPD patients.

Variables	Better sleep group (*n* = 94)	Low efficiency-maintenance group (*n* = 106)	High hypnotic-use with daytime dysfunction group (*n* = 31)	Test statistics	*p*-value
Gender				*χ*^2^ = 10.399	0.005
Male	84 (89.4%)	78 (73.6%)	21 (67.7%)		
Female	10 (10.6%)	28 (26.4%)	10 (32.3%)		
Age				H = 9.193	0.01
≤70	39 (41.5%)	39 (36.8%)	8 (25.8%)		
71–79	45 (47.9%)	46 (43.4%)	12 (38.7%)		
≥80	10 (10.6%)	21 (19.8%)	11 (35.5%)		
Educational level				H = 2.814	0.245
Primary education and below	44 (46.8%)	61 (57.5%)	16 (51.6%)		
Junior high school or high school degree	45 (47.9%)	42 (39.6%)	15 (48.4%)		
Bachelor degree or above	5 (5.3%)	3 (2.8%)	0		
Marital status				F^1^ = 1.201	0.633
Married	93 (98.9%)	104 (98.1%)	30 (96.8%)		
The others	1 (1.1%)	2 (1.9%)	1 (3.2%)		
Occupational status				*χ*^2^ = 0.305	0.992
In service	9 (9.6%)	9 (8.5%)	3 (9.7%)		
Self-employed or farming	64 (68.1%)	72 (67.9%)	22 (71%)		
Retire	21 (22.3%)	25 (23.6%)	6 (19.4%)		
Residence					
Rural area	57 (60.6%)	61 (57.5%)	22 (71%)	*χ*^2^ = 1.81	0.413
Town	37 (39.4%)	45 (42.5%)	9 (29%)		
Medical insurance type				F^1^ = 2.056	0.740
Resident medical insurance	90 (95.7%)	101 (95.3%)	29 (93.5%)		
Employee medical insurance	3 (3.2%)	2 (1.9%)	1 (3.2%)		
At one’s own expense	1 (1.1%)	3 (2.8%)	1 (3.2%)		
Per capita monthly income (CNY)				*χ*^2^ = 2.734	0.26
≤3,000	33 (35.1%)	40 (37.7%)	16 (51.6%)		
>3,000	61 (64.9%)	66 (62.3%)	15 (48.4%)		
Smoking status				*χ*^2^ = 9.456	0.049
Yes	8 (8.5%)	5 (4.7%)	4 (12.9%)		
Never	10 (10.6%)	27 (25.5%)	5 (16.1%)		
Quit smoking	76 (80.9%)	74 (69.8%)	22 (71%)		
Alcohol consumption status				*χ*^2^ = 12.074	0.016
Yes	3 (3.2%)	12 (11.3%)	2 (6.5%)		
Never	21 (22.3%)	39 (36.8%)	10 (32.3%)		
Quit drinking	70 (74.5%)	55 (51.9%)	19 (61.3%)		
Years of diagnosis of COPD				*χ*^2^ = 1.861	0.399
≤5	37 (39.4%)	38 (35.8%)	8 (25.8%)		
>5	57 (60.6%)	68 (64.2%)	23 (74.2%)		
CAT score	19.46 ± 5.48	23.8 ± 6.29	24.65 ± 5.51	F^2^ = 16.88	<0.001
SEMCD6 score	5.25 ± 1.22	4.57 ± 1.14	4.29 ± 1.08	F^2^ = 15.573	<0.001

1 Fisher’s exact test.

2 one-way analysis of variance.

### 3.3 Disordered multiclass logistic regression analysis of potential sleep quality profiles in COPD patients

The key variables identified in univariate analysis were employed as independent variables, while the three possible sleep quality profiles in COPD patients were utilized as dependent variables for multivariate logistic regression analysis. Table 3 displays that the CAT score, self-efficacy score, and gender significantly influenced the potential profiles (*p* < 0.05).

**Table 3:** Logistic regression analysis of three potential profiles of sleep quality in COPD patients.

Variable	Class1 (control group) vs. Class2			Class2 (control group) vs. Class3			Class1 (control group) vs. Class3		
	*β*	OR (95%CI)	*p*	*β*	OR (95%CI)	*p*	*β*	OR (95%CI)	*p*
CAT score	0.089	1.093 (1.024 ~ 1.167)	0.007	0	1 (0.916 ~ 1.091)	0.992	0.089	1.093 (0.995 ~ 1.201)	0.065
SEMCD6 score	−0.385	0.681 (0.478 ~ 0.969)	0.033	−0.164	0.849 (0.558 ~ 1.292)	0.445	−0.549	0.578 (0.358 ~ 0.931)	0.024
Gender (Ref. Female)									
Male	−1.066	0.345 (0.12 ~ 0.987)	0.047	−1.007	0.365 (0.111 ~ 1.205)	0.098	−2.072	0.126 (0.032 ~ 0.495)	0.003
Age (Ref. ≥ 80)									
≤70	−0.246	0.782 (0.288 ~ 2.126)	0.63	−1.019	0.361 (0.113 ~ 1.157)	0.086	−1.265	0.282 (0.078 ~ 1.017)	0.053
71–79	−0.249	0.779 (0.294 ~ 2.066)	0.616	−0.944	0.389 (0.135 ~ 1.117)	0.079	−1.193	0.303 (0.091 ~ 1.015)	0.053
Smoking Status (Ref. quit smoking)									
Yes	−0.679	0.507 (0.128 ~ 2)	0.332	1.437	4.209 (0.842 ~ 21.039)	0.08	0.758	2.133 (0.421 ~ 10.805)	0.36
Never	0.304	1.356 (0.412 ~ 4.463)	0.617	−1.2	0.301 (0.06 ~ 1.523)	0.147	−0.896	0.408 (0.067 ~ 2.505)	0.333
Alcohol consumption status (Ref. quit drinking)									
Yes	2.007	7.439 (1.74 ~ 31.794)	0.007	−0.844	0.43 (0.07 ~ 2.645)	0.363	1.163	3.199 (0.393 ~ 26.01)	0.277
Never	0.53	1.699 (0.692 ~ 4.177)	0.248	−0.39	0.677 (0.194 ~ 2.368)	0.541	0.14	1.151 (0.304 ~ 4.348)	0.836

Class1 is the better sleep group, Class2 is the low efficiency-maintenance group, Class3 is the high hypnotic-use with daytime dysfunction group.

## 4 Discussion

### 4.1 Population heterogeneity exists in sleep quality of COPD patients

Three profiles were identified among COPD patients admitted to AECOPD based on their overall sleep status in the past month: better sleep group, low efficiency-maintenance group and high hypnotic-use with daytime dysfunction group. Better sleep group, representing 40.6% of the sample, is characterized by superior performance across all domains compared to the other categories, with minimal reliance on sleep drug. Demographic analysis revealed a predominance of males in this category, typically of younger age with better physiological function. Moreover, individuals in this group tend to have higher monthly incomes exceeding 3,000 yuan and possess a more advanced level of education. Consequently, these patients exhibit greater capacity and resources for acquiring health-related knowledge, enabling them to develop a comprehensive understanding of their condition and exhibit enhanced adherence to treatment regimens, resulting in well-managed disease and sleep symptoms. In contrast, 46% of patients were classified as exhibiting low efficiency-maintenance group. These patients exhibited prolonged sleep onset latency, minimal reliance on sleep aids, fair daytime functioning, and poor sleep quality. Their suboptimal performance in sleep duration and efficiency dimensions may be attributed to a higher prevalence of alcohol consumption within this cohort. Previous research has indicated that pre-sleep alcohol consumption is associated with reduced average oxygen saturation levels and decreased rapid eye movement sleep duration, exacerbating sleep disturbances in COPD patients (13). Healthcare providers should educate patients on the detrimental effects of alcohol consumption on health and sleep, encouraging abstinence. Notably, 13.4% of individuals were classified as belonging to the high hypnotic-use with daytime dysfunction group, with patients in this category using hypnotic medications an average of 2–3 times weekly. Their self-reported sleep quality remains poor despite being able to sustain longer and better quality sleep with good sleep efficiency. Moreover, this group exhibits elevated levels of daytime fatigue and sleepiness, with a higher prevalence of COPD patients diagnosed for over 5 years, indicating more severe lung function impairment and a greater symptom load. This aligns with the findings of Enz et al. (14), who suggested that daytime sleepiness in COPD patients may be linked to nighttime respiratory disorders and potential medication-induced daytime fatigue. Kocevska et al. (15) found that elderly people have reduced rapid eye movement sleep, start sleep earlier than young people, an increased incidence of waking up at night, and an increased frequency of napping during the day. Therefore, the factor of advanced age cannot be ignored.

### 4.2 Factors influencing sleep quality in COPD patients

#### 4.2.1 Gender

The study’s findings indicate a significantly higher likelihood of men being classified under the category of better sleep group compared to women, aligning with previous research by Park et al. (16). Furthermore, the investigation revealed that females with COPD exhibit diminished lung carbon and oxygen diffusion capacities and experience a more pronounced annual decline relative to their male counterparts (17). Moreover, when exposed to equivalent levels of tobacco, female smokers demonstrate elevated concentrations of cigarette byproducts in their lungs, such as polycyclic aromatic hydrocarbon adducts (18), leading to heightened airflow limitation and earlier onset of exacerbations (19). The mechanisms underlying the observed gender-specific disparities in cigarette responsiveness remain incompletely elucidated, potentially attributed to increased airway wall thickness among women with COPD, characterized by a reduced airway lumen diameter associated with augmented airway wall dimensions (20).

#### 4.2.2 CAT scores

The CAT score serves as a valuable metric for assessing sleep quality in individuals with chronic obstructive pulmonary disease (COPD) (21). Ding et al. (5) identified a significant correlation between sleep disturbances and CAT scores, indicating that patients with elevated CAT scores exhibited more pronounced dyspnea symptoms (19). Their findings imply that individuals categorized as poor sleepers displayed higher CAT scores compared to those classified as good sleepers, underscoring a direct relationship between the severity of COPD symptoms and sleep quality. Consequently, healthcare providers should meticulously consider the COPD-sleep association and include sleep quality assessments as part of symptom evaluation in COPD patients.

#### 4.2.3 SEMCD6 score

The study findings indicated that individuals with COPD exhibiting higher levels of self-efficacy were more likely to be classified under the category of better sleep group. This relationship can be attributed to the correlation between elevated self-efficacy and higher educational attainment among patients, which may enhance their ability to manage the disease through increased knowledge and cognitive skills. Self-efficacy serves as a key determinant of health-related behaviors, with individuals demonstrating greater self-efficacy being more inclined to adhere to regimens, engage in pulmonary rehabilitation exercises, and practice good sleep hygiene. The sustained adherence to these behaviors yields long-term benefits that gradually enhance overall sleep quality. Nonetheless, obstacles to self-management in COPD patients may include cognitive deficits and the absence of reinforcement mechanisms (22). Therefore, interventions should focus on enhancing patients’ disease-related cognitive frameworks, establishing a supportive “knowledge-belief-action” loop that reinforces successful experiences through strategies such as simplified documentation and ongoing social, medical, and familial support. These multifaceted interventions aim to bolster patients’ confidence in managing their condition effectively.

#### 4.2.4 Alcohol consumption status

This investigation revealed that alcohol intake played a significant role in distinguishing various categories of sleep quality potential among patients with COPD. Specifically, individuals who consumed alcohol were more inclined to fall into the low efficiency-maintenance group. A previous retrospective analysis (23) highlighted alcohol consumption as a potential factor contributing to reduced engagement in primary care services. Thus, alcohol consumption not only impacts sleep patterns in COPD patients at a physiological level but also serves as a barrier to effective health management. Apart from its effects on respiration and sleep continuity, alcohol consumption commonly leads to heightened fatigue in COPD patients (24), thereby substantially impeding their capacity and motivation to manage a chronic illness. This discovery introduces a novel perspective for clinical interventions aimed at enhancing sleep quality and promoting active participation in disease management by emphasizing comprehensive education on the hazards of alcohol and providing support for abstinence.

## 5 Limitations

Limitations of this study include the cross-sectional design’s inability to establish causal relationships between individual sleep profiles in COPD patients and influencing factors. Future research could benefit from longitudinal studies to elucidate interrelationships among variables. Moreover, the sample was conveniently drawn from a single hospital, predominantly comprising male participants, thus limiting the generalizability of findings. Additionally, the University of Pittsburgh Sleep Quality Questionnaire, relying on self-reported data from the past month, may be susceptible to recall bias. Furthermore, the absence of data on GOLD staging, medication usage, and comorbidities could introduce selection and information biases. In addition, this study did not exclude patients with sleep apnea, including obstructive sleep apnea (OSA), through objective examination. While the PSQI questionnaire assesses associated symptoms, the influence of these comorbidities on the categorization of sleep quality profiles remains uncertain. Subsequent research should integrate objective sleep monitoring to precisely delineate sleep issues in COPD patients.

## 6 Summary

This study utilized latent signature analysis to identify three distinct clinical phenotypes among COPD patients, characterized by varying sleep qualities: the “better sleep group,” the “low efficiency-maintenance,” and the “high hypnotic-use with daytime dysfunction group.” These phenotypes not only exhibit differences in sleep architecture but also demonstrate systematic variations in gender distribution, alcohol consumption, CAT score, and self-efficacy score. This highlights the heterogeneity and complexity of sleep issues in COPD patients. By transcending the conventional understanding of sleep problems in COPD, this classification framework offers novel perspectives for clinical practice, emphasizing the importance of considering population heterogeneity.

Tailoring interventions to the specific characteristics of each patient group is crucial. For individuals in the “low efficiency-maintenance,” interventions should focus on sleep hygiene education and nocturnal symptom management. Patients in the “high hypnotic-use with daytime dysfunction group” require evaluation of medication appropriateness, identification of comorbidities, and promotion of non-pharmacological interventions. These findings establish a theoretical basis for developing personalized sleep management pathways in COPD care, aiming to enhance overall quality of life and long-term prognosis for patients.

## Data Availability

The original contributions presented in the study are included in the article/supplementary material, further inquiries can be directed to the corresponding author.
